# Gut bacterial *O*-demethylation in microbiome-dependent drug response

**DOI:** 10.1080/29933935.2026.2709263

**Published:** 2026-07-27

**Authors:** Cheng-Kai Chiang, Braden S. Dibello, Joshua Park, Jess Rondinella, Qihao Wu

**Affiliations:** a Department of Pharmaceutical Sciences, University of Pittsburgh, Pittsburgh, PA, USA

**Keywords:** Drug metabolism, gut microbiota, *O*-demethylation, pharmacokinetics

## Abstract

The gut microbiota is recognized as an important contributor to drug metabolism and therapeutic variability. By directly transforming orally administered drugs, gut microbes can influence drug exposure, efficacy, and toxicity. Among these microbial reactions, *O*-demethylation of methoxy-containing drugs remains relatively underexplored, despite the prevalence of this structural motif in many therapeutic agents. Recent studies suggest that gut bacterial *O*-demethylation can alter parent-drug exposure and generate metabolites with distinct biological activities, with potential consequences for therapeutic efficacy and toxicity across clinical settings. In this mini-review, we discuss gut bacterial *O*-demethylation as a distinct mechanism in microbiome-dependent pharmacology, with emphasis on its chemical logic, microbial basis, pharmacokinetic and toxicologic consequences, and translational potential for microbiome-targeted interventions and biomarker development.

## 1. Introduction

Orally administered drugs are exposed to a microbial community whose density rises steeply along the gastrointestinal tract, from sparse colonization of the stomach and proximal small intestine to dense populations within the colon.^1^ ^,^ ^2^ Although many drugs are absorbed in the small intestine, where microbial diversity and abundance are relatively low,^3^ a fraction of parent drug can still reach the distal gut because of incomplete absorption, limited solubility or permeability, high oral dose, slow dissolution, intestinal transit, or formulation-dependent release.^3-5^ In addition, absorbed drugs and host-generated metabolites can be returned to the intestinal lumen through biliary secretion.^6^ ^,^ ^7^ Together, these routes expose distal gut bacteria to parent drugs and drug-derived metabolites, where microbial reactions such as reduction, hydrolysis, deconjugation, decarboxylation, and other processes can overlap with or complement host metabolic pathways.^4^ ^,^ ^8-10^ These microbial transformations can change the amount of parent drug available for absorption, generate metabolites with altered biological activity, and contribute to interindividual variability in drug efficacy and toxicity.^5^ ^,^ ^11-13^ As a result, the gut microbiota is viewed as an additional metabolic site that can shape oral drug disposition and therapeutic response through a repertoire of chemical reactions whose pharmacological consequences remain only partially understood.^4^ ^,^ ^8^ ^,^ ^9^ ^,^ ^14^ ^,^ ^15^

Despite substantial progress in mapping microbiome-dependent drug metabolism, several microbial reaction types remain underexplored from a pharmacological perspective.^4^ One such reaction is *O*-demethylation, in which a methoxy group is converted to a hydroxyl group. This seemingly modest chemical change can have substantial consequences for drug behavior. *O*-demethylation can potentially alter polarity, hydrogen-bonding capacity, redox properties, receptor interactions, membrane permeability, and susceptibility to downstream host or microbial metabolism.^13^ ^,^ ^16-18^ Because aromatic methoxy groups are present in many clinically used drugs, gut bacterial *O*-demethylation may represent a broader source of microbiome-dependent drug metabolism than previously recognized.

The clinical relevance of this chemistry likely extends beyond a single drug or therapeutic area. Aryl methoxy groups are common across drugs used in oncology, cardiovascular disease, neurology, infectious disease, gastrointestinal disease, urology, and immune-related disorders^16^; thus, *O*-demethylation could affect a wider range of agents than currently established. The pharmacokinetic relevance of this reaction has been demonstrated most clearly for oral etoposide, whereas its impact on most other methoxy-containing drug classes remains to be established. For some drugs, microbial *O*-demethylation may reduce parent-drug exposure and compromise efficacy; for others, it may generate metabolites with enhanced activity, altered toxicity, or new off-target effects.^13^ ^,^ ^16^ Understanding when this reaction is pharmacologically meaningful will require moving beyond taxonomic associations toward functional biomarkers, enzyme-level mechanisms, and patient-specific measurements of microbial metabolic activity.

In this mini-review, we discuss gut bacterial *O*-demethylation as a potentially important but still underexplored mechanism of microbiome-dependent pharmacology. We highlight recent studies showing how this reaction can influence drug structure, metabolite bioactivity, pharmacokinetics, and toxicologic outcomes. We further discuss key translational challenges, including metabolite validation, identification of responsible microbes and enzymes, biomarker development, and functional assays to evaluate patient-specific microbial drug metabolism.

## 2. Broad microbiome-drug screens reveal candidate *O*-demethylation chemistry

**Figure 1.**
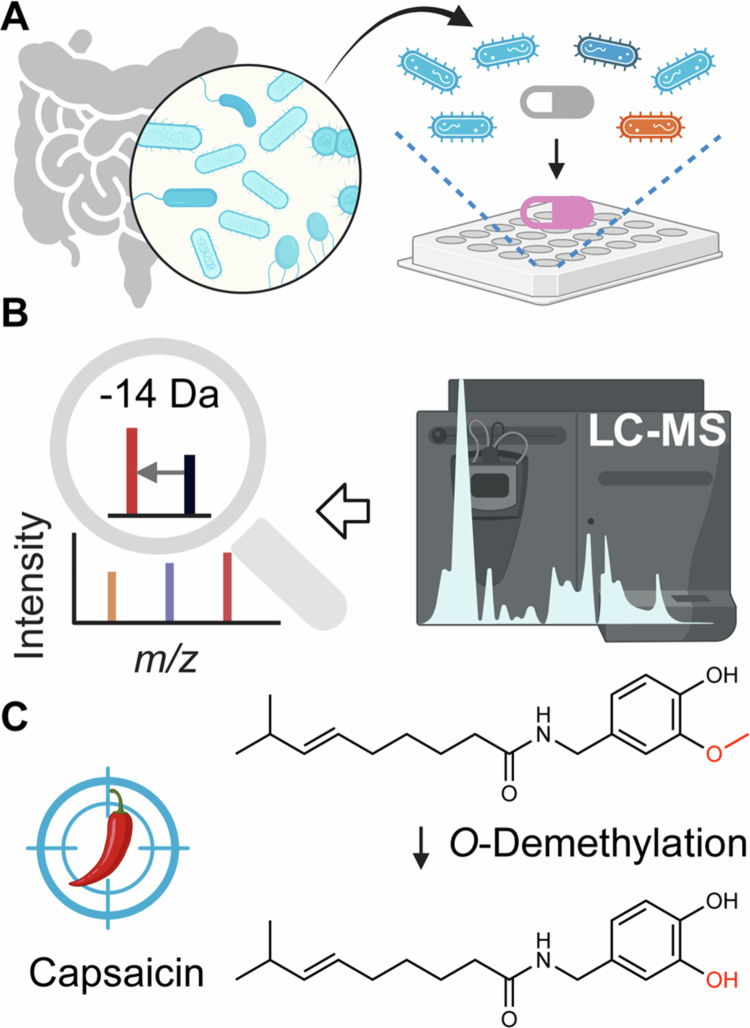
Broad microbiome-drug metabolism screens reveal candidate *O*-demethylation chemistry. (**A**) Large-scale *ex vivo* screening of diverse human gut bacterial strains against orally administered drugs showed that many drugs, including compounds with methoxy-containing aromatic scaffolds, are susceptible to gut bacterial metabolism. (**B**) LC-MS-based metabolomics analyzes can identify candidate demethylated products based on characteristic mass shifts. A mass loss of approximately 14 Da is chemically consistent with demethylation and may indicate *O*-demethylation when the substrate contains a methoxy group. (**C**) A representative case is capsaicin, for which a predicted product showed a mass shift consistent with possible *O*-demethylation. However, authentic standard comparison, tandem mass spectrometry (MS/MS), nuclear magnetic resonance (NMR) spectroscopy, and targeted microbial studies are still needed to confirm that this product indeed results from gut bacterial *O*-demethylation. Created with BioRender.com.

Systematic microbiome-drug metabolism screens show that *O*-demethylation is part of the broader chemical repertoire of the gut microbiota (Figure 1). Zimmermann et al.^8^ screened 76 phylogenetically diverse human gut bacterial strains against 271 orally administered drugs under anaerobic conditions, generating more than 20,000 bacteria-drug measurements by liquid chromatography-mass spectrometry (LC-MS) (Figure 1A and B). The drug library included multiple compounds bearing methoxy-containing aromatic scaffolds. Although the study did not focus on *O*-demethylation as a discrete reaction class, a mass loss of approximately 14 Da is chemically consistent with demethylation of methoxy-containing substrates, including potential *O*-demethylation. Thus, the Zimmermann screen provides broad evidence that methoxy-containing drugs are susceptible to gut bacterial metabolism and shows that targeted structural validation is needed to distinguish *O*-demethylation from other demethylation or dealkylation reactions.^8^

A complementary community-level screen by Javdan and colleagues further supports this view.^9^ Rather than testing isolated strains, this study developed Microbiome-Derived Metabolism-Screen, an *ex vivo* platform that uses subject-personalized gut microbial communities to assess microbiome-derived metabolism of orally administered drugs (Figure 1A and B). The structurally characterized transformations emphasized reduction, hydrolysis, deglycosylation, deacetylation, thioester hydrolysis, nitroreduction, and *N*-acetylation, rather than *O*-demethylation. However, capsaicin, an aryl methoxy-containing natural xenobiotic, was associated with predicted metabolites annotated as demethylation and demethylation plus reduction, including a −14.02 Da mass shift (Figure 1C). While the capsaicin metabolite assignment was based on high-resolution mass spectrometry (HRMS), this example suggests possible gut microbial *O*-demethylation, but structural confirmation will be needed.

## 3. *O*-demethylation can alter pharmacological activity

**Figure 2.**
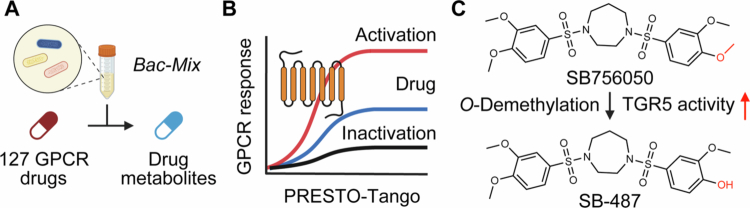
Activity-guided metabolomics links gut bacterial *O*-demethylation to altered GPCR drug activity. (**A**) A library of 127 GPCR-targeted drugs was incubated with the defined human gut bacterial community *Bac-Mix* to identify microbial drug metabolites. (**B**) PRESTO-Tango GPCR assays were used to determine whether microbial metabolism altered receptor activity, revealing metabolites that could increase, preserve, or reduce GPCR responses relative to the parent drug. (**C**) SB756050 was converted by gut bacterial *O*-demethylation to SB-487, a major *O*-demethylated metabolite with increased TGR5 agonist activity. This example illustrates that microbial *O*-demethylation can generate drug metabolites with altered, and in some cases enhanced, biological activity. Created with BioRender.com.

A more recent activity-guided screen further shows that gut bacterial *O*-demethylation can generate metabolites with altered biological activity.^13^ In a systematic comparative metabolomics study of 127 G protein-coupled receptor (GPCR)-targeted drugs exposed to a defined 30-member human gut bacterial community, 30 drugs were metabolized and 12 were robustly depleted (Figure 2A). Among the most extensively metabolized drugs, the study connected metabolite formation to receptor activity using PRESTO-Tango assays,^19^ ^,^ ^20^ revealing that gut microbial metabolism could inactivate, preserve, or enhance GPCR drug activity depending on the structural motif being modified (Figure 2B). Notably, *O*-demethylation was observed for drugs SB756050, trimethobenzamide, and trimebutine, and additional targeted analysis of 14 aryl *O*-methyl-containing GPCR drugs identified bacterial-cell-dependent formation of two predicted *O*-demethylated doxazosin metabolites. For SB756050, several *O*-demethylated products were detected, and the major metabolites were isolated and structurally confirmed by NMR. The resulting metabolites showed greater Takeda G protein-coupled receptor 5 (TGR5) agonist activity than the parent compound (Figure 2C). Similarly, microbial metabolism of trimethobenzamide was associated with increased GPCR activity. These findings suggest that, in selected cases, microbial *O*-demethylation can produce metabolites capable of altering receptor activity. However, the responsible bacterial member(s) for SB756050, trimethobenzamide, and trimebutine *O*-demethylation could not be assigned from the monoculture screen, leaving a major gap between community-level metabolic detection and taxon- or enzyme-level mechanism.

## 4. Microbial *O*-demethylation links drug metabolism to pharmacokinetics and toxicity

Recent work has begun to bridge this gap, including a study by Jeong and colleagues on the oral anticancer drug etoposide that identified gut bacterial taxa capable of *O*-demethylation and linked this activity to drug exposure and toxicity.^16^ Etoposide is a methoxy-containing anticancer drug with known variability in oral bioavailability and clinical response. Gut microbial *O*-demethylation converts etoposide to an *O*-demethylated metabolite with biological properties distinct from the parent compound. This transformation can reduce systemic exposure to orally administered etoposide while increasing exposure to the microbial metabolite, supporting the concept that gut bacteria can act as a pre-systemic metabolic barrier for selected oral drugs. Their study establishes pharmacokinetic relevance for etoposide and raises the possibility that gut bacterial *O*-demethylation is a broader route of oral drug metabolism than previously recognized.

**Figure 3.**
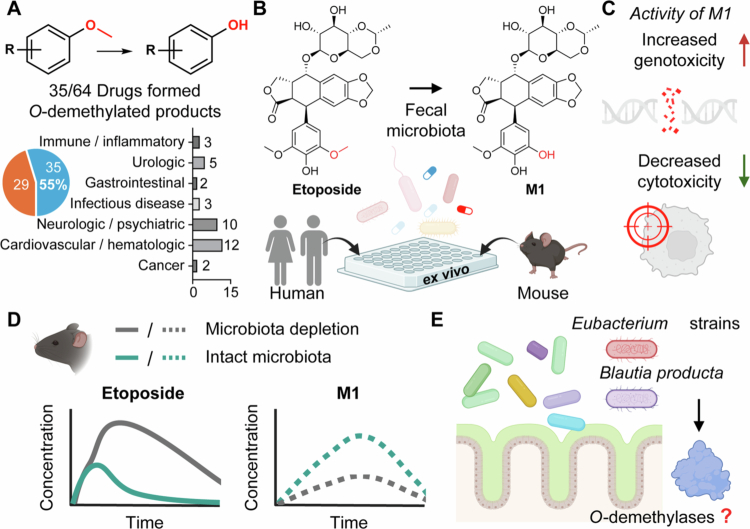
Gut bacterial *O*-demethylation is a widespread and pharmacologically important contributor to oral etoposide metabolism. (**A**) Screening of 64 clinically used oral drugs containing aromatic methoxy groups identified *O*-demethylated products for 35 compounds, indicating that gut microbial *O*-demethylation extends across drugs used in multiple therapeutic areas. (**B**) Etoposide is converted by human and mouse fecal microbiota *ex vivo* into the major *O*-demethylated metabolite M1, identified as etoposide catechol. (**C**) Relative to the parent drug, M1 displays altered biological activity, with decreased cytotoxicity but increased *in vivo* genotoxicity. (**D**) Depletion of the gut microbiota increases systemic exposure to orally administered etoposide while reducing exposure to M1, consistent with a pre-systemic role for gut microbes in shaping oral drug disposition. (**E**) Etoposide *O*-demethylation is distributed across multiple gut bacterial taxa, including several *Eubacterium* strains and *Blautia producta*, although the *O*-demethylase(s) remain unresolved. Created with BioRender.com.

To examine how widespread this activity is, the authors screened 64 clinically used oral drugs possessing aromatic methoxy groups and found *O*-demethylated products for 35 of them in mouse cecal-content assays. These findings suggest that gut microbial communities have broad *ex vivo* capacity to *O*-demethylate methoxy-containing drugs. This broad substrate range suggests that gut bacterial *O*-demethylation may influence drugs used across diverse therapeutic areas, including cancer, cardiovascular, neurologic, and infectious diseases (Figure 3A).

Jeong and colleagues show that gut microbiota convert etoposide into a major metabolite, M1, which they identify as etoposide catechol (Figure 3B). Furthermore, when etoposide was incubated *ex vivo* with unaltered mouse cecal contents or fresh human stool samples under anaerobic conditions, M1 formation was observed; however, this was not observed after boiling or antibiotic treatment, supporting a direct role for living gut bacteria in the reaction. These *ex* vivo incubations establish that gut bacteria are capable of *O*-demethylating etoposide, but on their own do not show whether the reaction proceeds at a pharmacologically meaningful scale in the gut.

Importantly, M1 displayed biological activities distinct from those of the parent drug. When compared with etoposide, M1 showed reduced cytotoxicity in cancer cell assays, suggesting diminished antitumor activity *in vitro*. In contrast, M1 displayed increased genotoxicity *in vivo*. This difference indicates that the consequences of microbial metabolism cannot be inferred from culture assays alone, since gut microbial metabolism may alter drug response in more than one way by reducing systemic parent-drug exposure and generating metabolites with a different toxicity profile (Figure 3C). For etoposide, a drug already linked to therapy-related secondary leukemia, this observation adds a particularly important clinical dimension to the study.

In pharmacokinetic experiments, depletion of the gut microbiota with antibiotics increased systemic exposure to etoposide while decreasing exposure to M1 (Figure 3D). In contrast, intravenous etoposide pharmacokinetics were largely unchanged. These data indicate that the gut microbiota influences oral etoposide disposition before systemic absorption and constitutes an important pre-systemic metabolic barrier. This framework establishes microbial metabolism of etoposide as a pharmacokinetically relevant contributor to host drug exposure, as a proof of principle supporting further exploration of gut microbial drug metabolism. The intravenous comparison additionally separates the microbial interaction from host intestinal and hepatic metabolism. By pairing *ex vivo* and *in vitro* assays with *in vivo* pharmacokinetic experiments, and by showing that intravenous pharmacokinetics were unchanged, the study distinguishes microbial contribution to oral drug exposure from the simple capacity for *O-*demethylation to occur in culture.

The study also identifies several gut bacterial species capable of etoposide *O*-demethylation. Etoposide *O*-demethylation is distributed across multiple gut bacterial species, including several *Eubacterium* strains and *Blautia producta* (Figure 3E). The results suggest that the capacity for this reaction is not confined to a rare microbe but may be distributed across the broader gut microbial community. This broader distribution has important implications for interindividual variability. Variation in the abundance of *O*-demethylation-capable taxa across individuals suggests that microbial community composition may be an overlooked contributor to differences in oral etoposide disposition and, potentially, treatment outcome.

However, the enzymatic basis of *O*-demethylation remains unresolved. Although the authors investigated candidate genes related to known *O*-demethylation systems, no single gene fully accounted for etoposide conversion. This unresolved mechanism defines an important next step for the field. As microbiome pharmacology advances, the ability to move from community-level metabolism to precise enzyme-level prediction will be critical for mechanistic understanding and future biomarker development.

## 5. Dietary and host-derived aryl methyl ethers in microbial *O*-demethylation

Although this mini-review focuses on drugs, gut bacterial *O*-demethylation has been more extensively described for non-pharmaceutical aryl methyl ethers, including diet-derived xenobiotics and host-derived metabolites.^17^ ^,^ ^18^ ^,^ ^21^ Many plant specialized metabolites contain aryl methoxy groups, and at least a subset of these compounds can reach the colon after incomplete absorption in the upper gastrointestinal tract, where they are exposed to anaerobic gut microbes.^22-25^ These examples provide useful biological context for considering how methoxy-containing drugs may also become substrates for gut microbial *O*-demethylation.

Bess and colleagues dissected a four-species bacterial consortium that converts the plant lignan pinoresinol into enterodiol and enterolactone.^17^ This pathway involves multiple coordinated reactions, including benzyl ether reduction, guaiacol demethylation, catechol dehydroxylation, and diol lactonization. Importantly, *Blautia producta* DSM3507 was implicated in demethylating the 3-position methoxy groups of secoisolariciresinol to generate didemethylsecoisolariciresinol, thereby forming catechol groups that enable downstream enterolignan production. This study connected *O*-demethylation to defined bacterial consortia, strain-level variation, inducible gene expression, human stool metabolism, and gnotobiotic validation, establishing dietary aryl methyl ether metabolism as a mechanistically tractable gut microbial function.

Host-derived methylated metabolites provide a complementary example in which gut bacterial *O*-demethylation intersects with host metabolism. Rich and colleagues showed that human fecal bacterial communities can *O*-demethylate 3-methoxytyramine, a catechol *O*-methyltransferase product of dopamine, back to dopamine.^18^ This activity was detected in fecal communities from four of seven human donors, suggesting interpersonal variation in the capacity to reverse host *O*-methylation. The authors further identified the acetogenic gut bacteria *Eubacterium limosum* and *Blautia producta* as organisms capable of this transformation: *E. limosum* DSM20543 quantitatively converted 3-methoxytyramine to dopamine, whereas two *B. producta* strains showed partial and strain-dependent conversion. Propyl iodide inhibited this reaction and light exposure partially reversed inhibition, supporting the involvement of cobalamin-dependent *O*-demethylases. The reaction was also associated with increased acetate production, linking aryl *O*-demethylation to reductive acetogenesis. Thus, gut bacterial *O*-demethylation can process both dietary xenobiotics and host-derived metabolites, and in some cases can regenerate a bioactive molecule from a host-methylated precursor.

A broader dietary xenobiotic screen by Culp and colleagues further places this chemistry in a community-level context.^21^ This study systematically mapped interactions between the gut microbiome and approximately 150 small-molecule dietary xenobiotics, including polyphenols, lignans, stilbenes, tannins, and related food-derived compounds. Using human fecal communities and defined bacterial communities, the authors showed that dietary xenobiotic metabolism varies substantially across individuals and can generate products through deglycosylation, ring opening, double-bond reduction, demethylation, and dehydroxylation. Notably, methoxylated polyphenols were metabolized by fewer communities than their non-methoxylated counterparts, suggesting that removal of methoxy groups is a specialized and variably distributed function. The study further noted that less common taxa, including acetogenic bacteria, can remove methoxyl groups from methoxylated polyphenols, likely through Wood-Ljungdahl-associated metabolism. These findings connect aryl *O*-methyl cleavage to interindividual variability, microbial community remodeling, and bioactivation or detoxification of dietary compounds.

Several other plant-derived phytochemicals can also undergo gut microbial *O*-demethylation, including isoflavones, citrus polymethoxyflavones, and methoxyphenolic acids.^26-28^ Although we do not discuss these examples in detail here, they further support the broader point that gut microbes can process *O*-methylated aromatic compounds commonly found in plant-rich diets.

Together, these dietary and host-metabolite examples provide a strong rationale for examining *O*-demethylation in drug metabolism. Gut bacterial *O*-demethylation likely did not evolve to process pharmaceuticals; rather, methoxy-containing drugs may be incidental substrates of microbial pathways that evolved to metabolize methylated plant specialized metabolites, dietary phenolics, and host-derived aryl methyl ethers. This perspective helps explain why *O*-demethylation is better established in the dietary and endogenous-metabolite literature than in the drug-metabolism literature. It also points to the central gap for pharmaceuticals, where broad drug screens and activity-guided studies can reveal *O*-demethylated drug metabolites and altered bioactivity, but in many cases the responsible taxa and enzymes remain unresolved. This gap indicates the need to connect candidate drug *O*-demethylation events to responsible microbes, enzymatic mechanisms, metabolite activity, and pharmacokinetic relevance.

## 6. Discussion

Gut bacterial *O*-demethylation represents a chemically simple transformation with potential pharmacological consequences. By converting aryl methoxy groups into phenolic or catecholic products, this reaction can alter drug polarity, redox properties, downstream metabolism, target engagement, toxicity, and systemic exposure. Evidence from dietary xenobiotics and host-derived metabolites shows that *O*-demethylation is a recurring gut microbial strategy for processing *O*-methylated aromatic compounds, whereas drug-focused studies are only beginning to define its scope and consequences. For methoxy-containing drugs, this reaction should therefore be viewed not as an isolated biochemical observation, but as a potentially important mechanism of microbiome-dependent pharmacology.

At present, the clinical translation of gut bacterial *O*-demethylation remains limited, largely because most substrates lack human pharmacokinetic data linking microbial metabolism to treatment outcomes. A major challenge is to distinguish detectable metabolism from clinically meaningful metabolism. Broad screens can reveal candidate *O*-demethylated products or demethylation-like mass shifts, but these observations require targeted validation through comparison with authentic standards, isotope tracing, MS/MS and NMR-based structural assignment, and controlled microbial models. Metabolite identification should be paired with functional testing, because *O*-demethylation may reduce parent-drug activity, enhance pharmacological activity, generate toxic or genotoxic products, or redirect a compound toward new biological targets. Future studies should therefore integrate chemical characterization with pharmacokinetic, pharmacodynamic, and toxicologic assays to determine whether these transformations explain clinically meaningful variability in drug response.

A related limitation is that much of the current evidence comes from *ex vivo* fecal, cecal, or defined-community assays. These systems are useful for identifying microbial metabolic capacity, but they do not fully capture the physiological constraints that determine whether *O*-demethylation occurs at a meaningful scale *in vivo.* ^29^ For any given drug, *in vivo* relevance is likely to depend on multiple factors, including the amount of parent compound or host-derived metabolite that reaches the distal gut, local concentration and residence time, and the regional distribution of responsible microbes. Host metabolism, biliary recycling, formulation properties, and other host- or microbiome-related variables may also influence whether *O*-demethylation has meaningful *in vivo* impact. Thus, detection of an *O*-demethylated product in culture should be interpreted as evidence of metabolic potential rather than direct evidence of pharmacological relevance. Establishing *in vivo* significance will require studies that pair *ex vivo* metabolism with pharmacokinetics, luminal or fecal metabolite measurements, controlled microbiota models, and functional assays at physiologically relevant concentrations.

Standardized validation platforms may help address the biological variability and labor-intensive preparation associated with raw fecal samples. Similar approaches have been used for other intestinal transformations; for example, variable human fecal enzyme preparations were replaced by a reproducible, freeze-dried Fecal Microbial Enzyme Mix to screen for dietary glycoside activation.^30^ ^,^ ^31^ An analogous standardized microbial or enzymatic panel enriched for *O*-demethylation activity could be useful for confirming microbiome-mediated demethylation chemistry, comparing substrates across studies, and prioritizing candidates for more detailed mechanistic work.

The field also needs to move from community-level observations to taxon- and enzyme-level mechanisms. Dietary and host-metabolite studies have shown that *O*-demethylation can be linked to defined bacteria, inducible gene programs, and cobalamin-dependent acetogenic metabolism. In contrast, for many drug substrates, the responsible bacterial species and enzymes remain unresolved. Identifying these microbial and enzymatic features will be essential for predicting which patients have high or low *O*-demethylation capacity and for understanding why this activity varies across individuals. Strain-level profiling, functional metagenomics, transcriptomics, gnotobiotic models, and enzyme biochemistry will be particularly important for this next phase.

Clinically, the most immediate opportunity is biomarker development rather than direct microbiome intervention. Stool metagenomics, targeted gene assays, *ex vivo* fecal metabolism assays, or LC-MS-based metabolite readouts could help identify patients whose microbiota *O*-demethylate selected oral drugs. Such biomarkers could eventually inform dose optimization, route selection, therapeutic drug monitoring, or toxicity monitoring. Formulation and drug-design strategies may provide complementary approaches to mitigate presystemic microbial metabolism, for example by limiting distal gut exposure, altering release profiles, selecting non-oral routes when appropriate, or modifying susceptible aryl methoxy motifs when pharmacologically feasible.^32^ Microbiome-targeted interventions, including diet, selective antibiotics, probiotics, defined consortia, or fecal microbiota transplantation, may become relevant in the future, but only after the responsible microbes, enzymes, and clinical consequences are established.

Overall, gut bacterial *O*-demethylation provides a useful framework for connecting microbial chemistry to precision pharmacology. The key question is no longer simply whether gut microbes can *O*-demethylate drugs, but when this reaction changes drug exposure, efficacy, or toxicity. Answering this question will require moving from broad metabolic discovery to mechanism-guided, patient-relevant models that connect drug structure, microbial function, metabolite activity, and therapeutic outcome.
